# Classification and characteristics of Japanese patients with antineutrophil cytoplasmic antibody-associated vasculitis in a nationwide, prospective, inception cohort study

**DOI:** 10.1186/ar4550

**Published:** 2014-04-23

**Authors:** Ken-ei Sada, Masahiro Yamamura, Masayoshi Harigai, Takao Fujii, Hiroaki Dobashi, Yoshinari Takasaki, Satoshi Ito, Hidehiro Yamada, Takashi Wada, Junichi Hirahashi, Yoshihiro Arimura, Hirofumi Makino

**Affiliations:** 1 Department of Medicine and Clinical Science, Okayama University Graduate School of Medicine, Dentistry and Pharmaceutical Sciences, 2-5-1 Shikata-cho, Kita-ku, Okayama 700-8558, Japan; 2 Center for Rheumatology, Okayama Saiseikai General Hospital, 1-17-18 Ifuku-cho, Kita-ku, Okayama 700-8511, Japan; 3 Department of Pharmacovigilance and Department of Medicine and Rheumatology, Graduate School of Medical and Dental Sciences, Tokyo Medical and Dental University, 1-5-45 Yushima, Bunkyo-ku, Tokyo 113-8519, Japan; 4 Department of Rheumatology and Clinical Immunology, Graduate School of Medicine, Kyoto University, 54 Shogoin-Kawahara-cho, Sakyo-ku, Kyoto 606-8507, Japan; 5 Division of Endocrinology and Metabolism, Hematology, Rheumatology and Respiratory Medicine, Department of Internal Medicine, Faculty of Medicine, Kagawa University, 1750-1 Ikenobe, Miki-cho, Kita-gun, Kagawa 761-0793, Japan; 6 Department of Internal Medicine and Rheumatology, Juntendo University School of Medicine, 2-1-1 Hongo, Bunkyo-ku, Tokyo 113-8431, Japan; 7 Department of Internal Medicine, Faculty of Medicine, University of Tsukuba, 1-1-1 Tennodai, Tsukuba, Ibaraki 305-8575, Japan; 8 Division of Rheumatology and Allergology, Department of Internal Medicine, St. Marianna University School of Medicine, 2-16-1 Sugao, Miyamae-ku, Kawasaki 216-8511, Japan; 9 Division of Nephrology, Department of Laboratory Medicine, Institute of Medical, Pharmaceutical and Health Sciences, Faculty of Medicine, Kanazawa University, 13-1 Takara-machi, Kanazawa 920-8641, Japan; 10 Department of Nephrology and Endocrinology, Graduate School of Medicine, The University of Tokyo, 7-3-1 Hongo, Bunkyo-ku, Tokyo 113-8655, Japan; 11 First Department of Internal Medicine, Kyorin University School of Medicine, 6-20-2 Shinkawa, Mitaka, Tokyo 181-8611, Japan

## Abstract

**Introduction:**

We investigated the clinical and serological features of patients with antineutrophil cytoplasmic antibody (ANCA)-associated vasculitis (AAV) in Japan using data from a nationwide, prospective, inception cohort study.

**Methods:**

In total, 156 Japanese patients with newly diagnosed AAV were classified according to the European Medicines Agency (EMEA) algorithm with exploratory surrogate markers for AAV-related non-granulomatous pulmonary lesions, predefined as alveolar haemorrhage and interstitial lung disease (ILD), and their clinical and serological features were evaluated.

**Results:**

Using the EMEA algorithm, we identified 14 patients (9.0%) with eosinophilic granulomatosis with polyangiitis (EGPA), 33 (21.2%) with granulomatosis with polyangiitis (GPA), 78 (50.0%) with microscopic polyangiitis and renal-limited vasculitis (MPA/RLV), and 31 (19.9%) with unclassifiable vasculitis. The average ages of patients with EGPA (male/female, 5/9), GPA (12/21), and MPA/RLV (35/43) and unclassifiable (9/22) were 58.0, 63.6, 71.1, and 70.6 years, respectively. Myeloperoxidase (MPO)-ANCA and proteinase-3 ANCA positivity was 50.0% and 0% for EGPA, 54.6% and 45.5% for GPA, 97.4% and 2.6% for MPA/RLV, and 93.5% and 3.2% for unclassifiable, respectively. According to the Birmingham Vasculitis Activity Score (BVAS), cutaneous (71.4%) and nervous system (92.9%) manifestations were prominent in EGPA and ear, nose, and throat manifestations (84.9%) and chest manifestations (66.7%) in GPA. Renal manifestations developed frequently in MPA/RLV (91.0%) and GPA (63.6%). The average serum creatinine levels were 0.71 mg/dL for EGPA, 1.51 mg/dL for GPA, 2.46 mg/dL for MPA/RLV, and 0.69 mg/dL for unclassifiable. The percentages of patients with ILD were 14.3% for EGPA, 9.0% for GPA, 47.4% for MPA/RLV, and 61.3% for unclassifiable. Patients with ILD (*n* = 61) had significantly lower BVAS (*P* = 0.019) with fewer ear, nose, and throat and cardiovascular manifestations than patients without ILD (*n* = 95).

**Conclusions:**

MPO-ANCA-positive MPA/RLV is the most common form of AAV in Japanese patients, and one-half of patients with GPA were positive for MPO-ANCA. ILD is an important clinical manifestation in Japanese patients with AAV. Unclassifiable vasculitis with MPO-ANCA positivity and ILD may represent a novel variant of MPA.

**Trial Registration:**

The University Hospital Medical Information Network Clinical Trials Registry: UMIN000001648. Registered 28 February 2009.

## Introduction

Microscopic polyangiitis (MPA), granulomatosis with polyangiitis (Wegener’s granulomatosis) (GPA), and eosinophilic granulomatosis with polyangiitis (Churg–Strauss syndrome) (EGPA) are the major categories of antineutrophil cytoplasmic antibody (ANCA)-associated vasculitis (AAV), a multisystem autoimmune disease characterised by ANCA production and small-vessel inflammation [1,2]. Despite the overlapping clinicopathologic characteristics between the component diseases, the disease evolution, organ involvement, prognosis, and other clinical characteristics differ substantially among them. In addition, there are interesting geographic and ethnic differences in their relative incidence and myeloperoxidase (MPO)-ANCA or proteinase-3 (PR3)-ANCA positivity [3].

In 2007 Watts and colleagues proposed an AAV classification algorithm, the European Medicines Agency (EMEA) algorithm, with consensus of a group of European physicians interested in the epidemiology of vasculitis [4]. This stepwise algorithm incorporated both the American College of Rheumatology (ACR) criteria for EGPA and GPA and the Chapel Hill Consensus Conference (CHCC) definition of EGPA, GPA, and MPA [2]. In the EMEA algorithm, surrogate markers of granulomatous inflammation for GPA and those of renal vasculitis for renal-limited vasculitis (RLV), an organ-limited variant of MPA, were defined [4]. This algorithm is useful for classifying patients with AAV because no overlapping diagnoses occur and fewer patients are considered to have unclassifiable vasculitis [5], and has been used as the standard method for classification of AAV diseases in recent studies [6,7].

Only two reports have validated the algorithm in other ethnicities outside Europe using a good-quality database. Studies from China [5] and Japan [3] applied the EMEA algorithm to their patient populations and found that MPO-ANCA-positive MPA was the most common form of AAV. These studies, however, were retrospective and evaluated clinical data of patients from a small number of hospitals.

The lung is one of the organs frequently involved in AAV, and pulmonary granuloma, alveolar haemorrhage, and interstitial lung disease (ILD) are representative pulmonary lesions. Among these, only pulmonary granuloma is included in the EMEA algorithm. ILD in AAV is associated with MPO-ANCA and is more common in Asian countries [8-10] than in western countries [11,12], and some patients with MPO-ANCA and ILD subsequently develop typical MPA [13]. To understand the nature of AAV and classify the disease from a global perspective, it is essential to more precisely delineate the clinical implications of ILD in AAV in Asian countries.

To characterise the clinical and laboratory features, effectiveness, and safety of the remission-induction therapy used, as well as the prognosis of Japanese patients with AAV, the Research Committee on Intractable Vasculitides of the Ministry of Health, Labour and Welfare of Japan implemented a nationwide prospective cohort study of Remission Induction Therapy in Japanese Patients with ANCA-associated Vasculitides (RemIT-JAV). In this study, we classified Japanese patients with newly diagnosed AAV enrolled in the RemIT-JAV study according to the EMEA algorithm and compared their phenotypes across the AAVs. We also investigated the clinical relevance of ILD in the patient population.

## Methods

### Database

Twenty-two tertiary care institutions (university hospitals and referring hospitals) participated in this study (See Appendix) and enrolled consecutive patients with newly diagnosed AAV from April 2009 to December 2010. The criteria for enrolment in this study included receiving a diagnosis of AAV from the site investigators, fulfilling the criteria for primary systemic vasculitis proposed by the EMEA algorithm [4], and requiring immunosuppressive treatment based on the discretion of the site investigators. The exclusion criteria were age younger than 20 years, recurrent AAV, serological evidence for hepatitis B virus or hepatitis C virus infection, and a history of malignancies because this may influence treatment selection and prognosis of patients with AAV. We conducted this study according to the Declaration of Helsinki and the Ethical Guidelines for Epidemiological Research in Japan. Written informed consent was obtained from each participant, and the study protocol was approved by the ethics committee at each participating hospital (refer to Acknowledgements). This study was registered with the University Hospital Medical Information Network Clinical Trials Registry (UMIN000001648).

### Data collection

Each patient’s baseline data included demographic information, general performance categorised using scales of the World Health Organization performance status except category 5 (death) [14], comorbidities, laboratory data, disease activity scored using the Birmingham Vasculitis Activity Score (BVAS) 2003 [15], imaging data (for example, chest radiograph, thoracic computed tomography, and magnetic resonance imaging of the head), and respiratory function data. The World Health Organization performance status runs from 0 to 5, with 0 denoting perfect health and 5 denoting death (0, asymptomatic; 1, symptomatic but completely ambulatory; 2, symptomatic, <50% in bed during the day; 3, symptomatic, >50% in bed, but not bedbound; 4, bedbound; 5, death).

Patients were evaluated at months 3, 6, 12, 18, and 24 and at relapse, and the following data were collected: vital status, BVAS 2003, laboratory data, treatments, and adverse events. The Vascular Damage Index score was recorded at months 6, 12, and 24. Chest radiography, thoracic computed tomography, arterial blood gas analysis, and respiratory function data were collected at months 12 and 24 in patients with pulmonary involvement. Observation was completed in March 2013. Only the baseline data are included in this study; the results from analyses of follow-up data will be reported separately.

The site investigators completed and sent the electronic case report form for each patient to the RemIT-JAV data centre at the Department of Medicine and Clinical Science, Okayama University Graduate School of Medicine, Dentistry and Pharmaceutical Sciences, Okayama, Japan.

### EMEA classification algorithm for AAV

The enrolled patients were classified using the stepwise EMEA algorithm as described previously [2,4]. Briefly, the ACR criteria and Lanham criteria for EGPA were applied first. Patients who did not fulfil the criteria for EGPA were classified as having GPA if they met the ACR criteria for GPA or the CHCC histological definition for GPA or if they showed histology compatible with the CHCC definition for MPA or ANCA positivity with either of the EMEA-defined GPA surrogate markers. The remaining patients were classified as having MPA if they had clinical features and histology compatible with small-vessel vasculitis without the GPA surrogate markers. In addition, ANCA-positive patients who had the EMEA-defined surrogate markers for renal vasculitis were classified as having RLV, a variant form of MPA. The rest of the patients without histology compatible with the CHCC definition of classic polyarteritis nodosa or typical angiographic features of classic polyarteritis nodosa were categorised as having unclassifiable vasculitis.

To identify a subset of unclassifiable vasculitis with AAV-related nongranulomatous pulmonary lesions, we defined exploratory surrogate markers for alveolar haemorrhage and ILD and then applied them to the EMEA-defined unclassifiable patient population. Surrogate markers for these conditions were as follows: haemoptysis or alveolar haemorrhage evaluated by bronchoscopic examination; or ILD diagnosed by chest X-ray or thoracic computed tomography.

### Disease severity

The disease severity of the enrolled patients was classified as localised, early systemic, generalised, or severe according to the European League Against Rheumatism recommendation for conducting a clinical study in systemic vasculitis [16]. Organ failure, classified as severe disease, was defined by the presence of any of the following BVAS manifestations: massive haemoptysis/alveolar haemorrhage, respiratory failure, congestive cardiac failure, ischaemic abdominal pain, or stroke. Threatened vital organ function, classified as generalised disease, was defined by the presence of any of the following BVAS manifestations: sudden visual loss, blurred vision, retinal changes (vasculitis/thrombosis/exudates/haemorrhage), conductive deafness, sensorineural hearing loss, ischaemic cardiac pain, cardiomyopathy, peritonitis, bloody diarrhoea, meningitis, organic confusion, seizures, cord lesion, cranial nerve palsy, sensory peripheral neuropathy, or motor mononeuritis multiplex. Serum creatinine levels were also used to classify disease severity as localised and early systemic (<120 μmol/l (1.3 mg/dl)), generalised (<500 μmol/l (5.5 mg/dl)), and severe (≥500 μmol/l (5.5 mg/dl)) [16].

### Statistical analysis

We used the baseline data of the patients enrolled in this study for statistical analysis. The primary purpose of this analysis was to determine the demographic and clinical characteristics of Japanese patients with AAV. Categorical variables were compared using Fisher’s direct probability test, and continuous variables were compared using Student *t* test or the Mann–Whitney *U* test depending on data distribution. *P* < 0.05 was considered significant for statistical analyses between two categories. When comparing among three categories, statistical significance was determined by *P* < 0.05/3 using Bonferroni correction to avoid multiplicity. All statistical analyses were performed by a biostatistician using the Statistical Package of JMP for Windows software (version 8.0.2; SAS Institute Inc., Cary, NC, USA).

## Results

### Classification of 156 Japanese patients with AAV according to the EMEA algorithm

In total, 159 patients with AAV were initially enrolled in the RemIT-JAV study. Three patients were then excluded; two patients did not undergo treatment, and one patient had been diagnosed as having AAV and experienced a relapse at the time of enrolment. As a result, 156 patients with newly diagnosed AAV were enrolled in the study.

**Figure 1:**
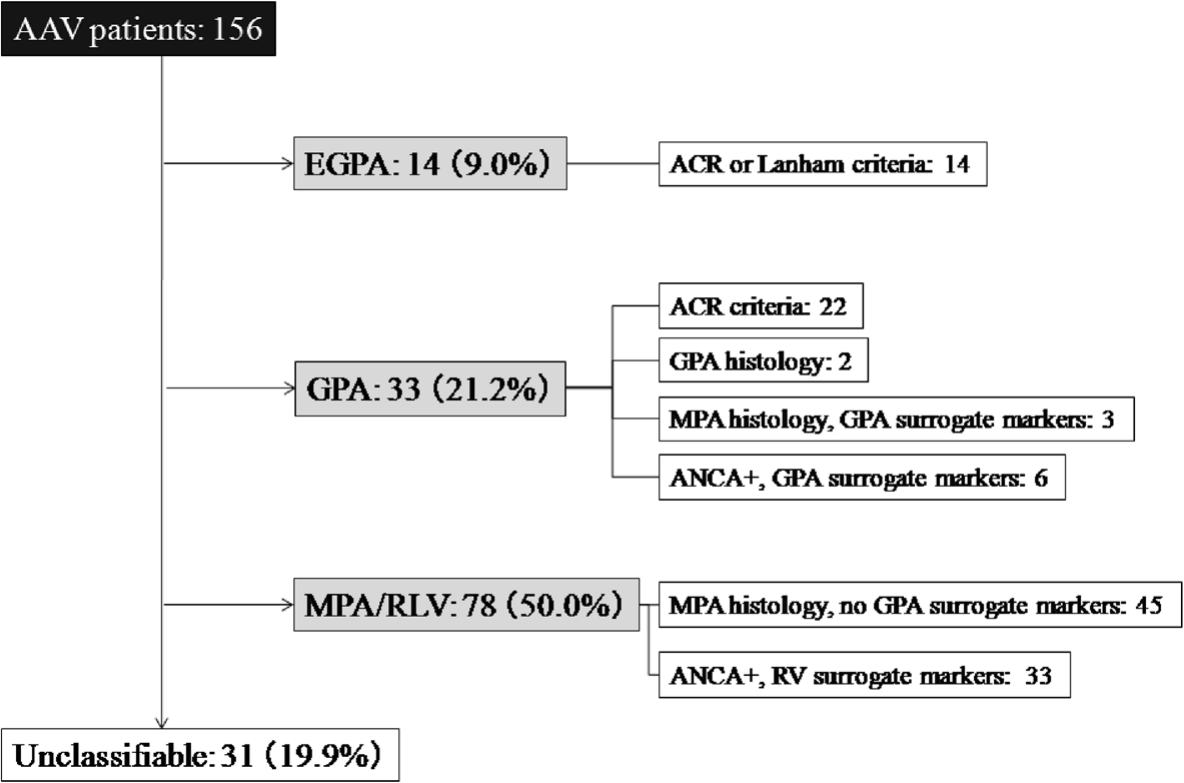
**Classification of patients with antineutrophil cytoplasmic antibody-associated vasculitis according to the EMEA algorithm in this cohort study.** AAV, antineutrophil cytoplasmic antibody-associated vasculitis; ACR, American College of Rheumatology; ANCA, antineutrophil cytoplasmic antibody; EGPA, eosinophilic granulomatosis with polyangiitis (Churg–Strauss syndrome); EMEA, European Medicines Agency; GPA, granulomatosis with polyangiitis (Wegener’s granulomatosis); MPA, microscopic polyangiitis; RLV, renal-limited vasculitis; RV, renal vasculitis.

Using the EMEA algorithm, we identified 14 patients with EGPA, 33 patients with GPA, 78 patients with MPA/RLV, and 31 patients who were unclassifiable (Figure 1). The average ages of the patients with EGPA (male/female, 5/9), GPA (male/female, 12/21), and MPA/RLV (male/female, 35/43) were 58.0, 63.6, and 71.1 years, respectively (Table 1). Patients with MPA/RLV were significantly older at the time of presentation than those with EGPA and GPA (*P* < 0.017 for both), and there was a female predominance for all AAV diseases. MPO-ANCA was detectable in 50.0% of patients with EGPA, in 54.6% of those with GPA, and in 97.4% of those with MPA/RLV. In contrast, PR3-ANCA was detectable in none of the patients with EGPA, in 45.5% of those with GPA, and in 2.6% of those with MPA/RLV.

**Table 1:** Comparison of demographics and disease states among AAV diseases

	**EGPA**	**GPA**	**MPA/RLV**	**Unclassifiable**
	**(*n*** **= 14)**	**(*n*** **= 33)**	**(*n*** **= 78)**	**(*n*** **= 31)**
Male/female	5/9	12/21	35/43	9/22
Mean (median) age (years)^a,c^	58.0 ± 16.9 (62)	63.6 ± 12.6 (61)	71.1 ± 10.0 (73)	70.6 ± 11.8 (73)
MPO-ANCA^a,c^	7 (50.0)	18 (54.6)	76 (97.4)	29 (93.5)
PR3-ANCA^b,c^	0 (0)	15 (45.5)	2 (2.6)	1 (3.2)
ANCA-negative^a,b^	7 (50.0)	3 (9.1)	1 (1.3)	2 (6.5)
Serum creatinine (mg/dl)^a^	0.71 ± 0.39	1.51 ± 1.32	2.46 ± 2.18	0.69 ± 0.23
Disease severity^c^				
Localised	0 (0)	4 (12.1)	0 (0)	0 (0)
Early systemic	1 (7.1)	5 (15.1)	15 (19.2)	15 (48.4)
Generalised	13 (92.9)	18 (54.6)	47 (60.3)	13 (41.9)
Severe	0 (0)	6 (18.2)	16 (20.5)	3 (9.7)
General performance^d^				
0/1/2/3/4	1/7/2/4/0	8/11/2/11/1	10/29/17/16/6	3/11/7/9/1
Nongranulomatous pulmonary involvement				
Interstitial lung disease^a^	2 (14.3)	3 (9.0)	37 (47.4)	19 (61.3)
Alveolar haemorrhage	0 (0)	2 (6.1)	9 (11.5)	2 (6.5)

Values expressed as mean ± standard deviation or number (percentage) unless otherwise noted. AAV, antineutrophil cytoplasmic antibody-associated vasculitis; ANCA, antineutrophil cytoplasmic antibody; EGPA, eosinophilic granulomatosis with polyangiitis (Churg–Strauss syndrome); GPA, granulomatosis with polyangiitis (Wegener’s granulomatosis); MPA, microscopic polyangiitis; MPO, myeloperoxidase; PR3, proteinase-3; RLV, renal-limited vasculitis. Comparisons between the EGPA, GPA, and MPV/RLV groups were made by Student *t* test or Mann–Whitney *U* test. Statistical significance was determined by *P* < 0.05/3 using Bonferroni correction: ^a^EGPA versus MPA/RLV, ^b^EGPA versus GPA, ^c^GPA versus MPA/RLV. Unclassifiable AAV was not compared with other forms of AAV. ^d^General performance was categorised according to the World Health Organization performance status except category 5 (death).

### Surrogate markers involved in the classification according to the EMEA algorithm

Of the patients classified with GPA, 22 patients fulfilled the ACR criteria, two patients showed CHCC-defined GPA histology, three patients showed CHCC-defined MPA histology in the presence of EMEA-defined GPA surrogate markers, and six patients were positive for ANCA in the presence of GPA surrogate markers. Consequently, nine patients were classified by the presence of GPA surrogate markers, mostly by the presence of chronic sinusitis or otitis media (Table 2). Of the patients classified with MPA/RLV, 45 patients showed histology compatible with small-vessel vasculitis without GPA surrogate markers and 33 patients were positive for ANCA with EMEA-defined surrogate markers for renal vasculitis. Three patients with allergic rhinitis, but not asthma, who had eosinophilia of peripheral blood and tissue were considered to have unclassifiable vasculitis. The eosinophilic vasculitis was confirmed histologically in all three patients, and two of these three patients were MPO-ANCA-positive. The exploratory surrogate markers for AAV-related nongranulomatous pulmonary lesions were positive in 16 of the 31 unclassifiable patients; three patients had both markers (alveolar haemorrhage and ILD), and 13 patients had only ILD. These 16 patients were all positive for MPO-ANCA.

**Table 2:** Surrogate markers in nine patients who were classified with granulomatosis with polyangiitis using these markers

	**Number of patients**
	**(MPO-ANCA/PR3-ANCA**^**a**^**)**
X-ray evidence of fixed pulmonary infiltrates, nodules, or cavitations present for >1 month	1 (1/0)
Bronchial stenosis	0 (0/0)
Bloody nasal discharge and crusting for 1 month or nasal ulceration	1^b^ (0/1)
Chronic sinusitis, otitis media, or mastoiditis for >3 months	7^c^ (4/5)
Retro-orbital mass or inflammation (pseudotumor)	0 (0/0)
Subglottic stenosis	1 (0/1)
Saddle nose deformity/destructive sinonasal disease	1 (0/1)

ANCA, antineutrophil cytoplasmic antibody; MPO, myeloperoxidase; PR3, proteinase-3. ^a^MPO-ANCA/PR3-ANCA, anti-myeloperoxidase antibody-positive versus anti-proteinase-3 antibody-positive patients. ^b^One patient had bloody nasal discharge and crusting and chronic sinusitis, and another patient had subglottic stenosis and saddle nose deformity. ^c^Five patients with chronic sinusitis, three patients with otitis media, and no patients with mastoiditis.

### Organ involvement of 156 Japanese patients with AAV

Patterns of organ involvement defined by the BVAS 2003 scoring system were described and compared among patients with EGPA, GPA, and MPA/RLV (Table 3). Most patients with AAV presented with constitutional symptoms. Cutaneous and nervous system manifestations were most common in patients with EGPA (71.4% and 92.9%, respectively). Ear, nose, and throat manifestations and chest manifestations were characteristic of patients with GPA (84.9% and 66.7%, respectively). It is noteworthy that ILD is not included in the BVAS 2003 scoring system. Renal manifestations developed frequently in patients with MPA/RLV (91.0%) but also in patients with GPA (63.6%). The mean serum creatinine level and prevalence of ILD in patients with MPA/RLV was higher than that in patients with EGPA and GPA, with a significant difference between MPA/RLV and EGPA for both (Table 1). Unclassifiable patients had cutaneous (32.3%), renal (48.4%), and nervous system (48.4%) manifestations (Table 3).

**Table 3:** Comparison of disease activity and organ involvement among AAV diseases

	**EGPA**	**GPA**	**MPA/RLV**	**Unclassifiable**
	**(*n*** **= 14)**	**(*n*** **= 33)**	**(*n*** **= 78)**	**(*n*** **= 31)**
BVAS	16.1 ± 7.7	19.9 ± 7.4	18.4 ± 7.3	12.1 ± 7.6
General	10 (71.4)	23 (69.7)	56 (71.8)	29 (93.6)
Cutaneous^a,b^	10 (71.4)	3 (9.1)	16 (20.5)	10 (32.3)
Mucous membranes/eyes	1 (7.1)	8 (24.2)	9 (11.5)	2 (6.5)
Ear, nose, and throat^a,b,c^	6 (42.9)	28 (84.9)	7 (9.0)	1 (3.2)
Chest^c^	5 (35.7)	22 (66.7)	30 (38.5)	6 (19.4)
Cardiovascular	2 (14.3)	4 (12.1)	6 (7.7)	0 (0)
Abdominal	0 (0)	1 (3.0)	0 (0)	1(3.2)
Renal^a,b^	2 (14.3)	21 (63.6)	71 (91.0)	15 (48.4)
Nervous system^a,b^	13 (92.9)	14 (42.4)	33 (42.3)	15 (48.4)

Values expressed as mean ± standard error or number (percentage). Disease activity and patterns of organ involvement were defined by the BVAS 2003 scoring system. AAV, antineutrophil cytoplasmic antibody-associated vasculitis; BVAS, Birmingham Vasculitis Activity Score; EGPA, eosinophilic granulomatosis with polyangiitis; GPA, granulomatosis with polyangiitis (Wegener’s granulomatosis); MPA, microscopic polyangiitis; RLV, renal-limited vasculitis. Comparisons between the EGPA, GPA, and MPV/RLV groups were made by Student *t* test or Mann–Whitney *U* test. Statistical significance was determined by *P* < 0.05/3 using Bonferroni correction: ^a^EGPA versus GPA, ^b^EGPA versus MPA/RLV, ^c^GPA versus MPA/RLV. Unclassifiable AAV was not compared with other forms of AAV.

### Differences in clinical features between MPO-ANCA-positive and PR3-ANCA-positive AAV

We compared the demographic and clinical features of Japanese patients with AAV who had MPO-ANCA and those who had PR3-ANCA (Table 4). Patients with MPO-ANCA were significantly older at the time of presentation (*P* = 0.012) and had a higher rate of ILD (*P* = 0.0015). The mean serum creatinine level was numerically higher in patients with MPO-ANCA. According to the BVAS 2003 scoring system, MPO-ANCA-positive patients had more cutaneous (*P* = 0.046) and renal (*P* = 0.010) manifestations and fewer ear, nose, and throat manifestations (*P* < 0.0001) with statistical significance.

**Table 4:** Comparison of demographics and disease manifestations in MPO-ANCA-positive and PR3-ANCA-positive patients

	**MPO-ANCA**	**PR3-ANCA**	***P *** **value**
	**(*n*** **= 125)**	**(*n*** **= 13)**	
Male/female	47/78	6/7	0.56
Mean (median) age (years)	70.0 ± 1.04 (73)	61.3 ± 3.2 (61)	0.012
Serum creatinine (mg/dl)	1.94 ± 0.17	1.22 ± 0.53	0.19
Interstitial lung disease	57 (45.6)	0 (0)	0.0015
Alveolar haemorrhage	11 (8.8)	1 (7.7)	0.89
Disease severity			0.26
Localised	2 (1.6)	1 (7.7)	
Early systemic	33 (26.4)	2 (15.4)	
Generalised	68 (54.4)	8 (61.5)	
Severe	22 (17.6)	2 (15.4)	
General performance^a^			0.26
0/1/2/3/4	16/44/26/31/8	4/6/1/2/0	
BVAS^b^			
BVAS	17.5 ± 0.71	17.5 ± 2.2	0.99
General	95 (76.0)	10 (76.9)	0.94
Cutaneous	30 (24.0)	0 (0)	0.046
Mucous membranes/eyes	16 (12.8)	4 (30.8)	0.08
Ear, nose, and throat	22 (17.6)	12 (92.3)	<0.0001
Chest	49 (39.2)	7 (53.9)	0.31
Cardiovascular	8 (6.4)	2 (15.4)	0.23
Abdominal	2 (1.6)	0 (0)	0.65
Renal	98 (78.4)	6 (46.2)	0.010
Nervous system	55 (44.0)	4 (30.8)	0.36

Values expressed as mean ± standard error or number (percentage) unless otherwise noted. Five patients who were double-positive for both ANCAs were excluded from this analysis. ANCA, antineutrophil cytoplasmic antibody; BVAS, Birmingham Vasculitis Activity Score; MPO, myeloperoxidase; PR3, proteinase-3. ^a^General performance was categorised according to the World Health Organization performance status except category 5 (death). ^b^Disease activity and patterns of organ involvement were defined by the BVAS 2003 scoring system.

Of the 33 patients with GPA, 15 patients, 12 patients, and three patients were positive for MPO-ANCA alone, for PR3-ANCA alone, or for both ANCAs, respectively, but three patients were negative for ANCA. Patients with GPA who had MPO-ANCA had a numerically higher rate of renal disease (86.7%) than those with PR3-ANCA (41.7%). The mean serum creatinine level of patients with MPO-ANCA-positive GPA (2.05 ± 0.35 mg/dl) was also numerically higher than that of patients with PR3-ANCA-positive GPA (1.03 ± 0.39 mg/dl).

### Clinical features of patients with or without interstitial lung disease

We compared the demographic and clinical characteristics of the patients with and without ILD (Table 5). MPO-ANCA was found significantly more frequently (*P* < 0.001) and PR3-ANCA was found less frequently (*P* = 0.038) in patients with ILD. These patients also tended to have more early systemic diseases and less generalised or severe diseases (*P* = 0.059) and had significantly lower BVAS (*P* = 0.019). The mean serum creatinine level and rates of patients with constitutive symptoms were similar between the two subgroups. The patients with ILD also had statistically fewer ear, nose, and throat (*P* = 0.006) and cardiovascular (*P* = 0.012) manifestations.

**Table 5:** Comparing patients with or without interstitial lung disease

	**Patients with ILD**	**Patients without ILD**	***P *** **value**
	**(*n*** **= 61)**	**(*n*** **= 95)**	
Male/female	28/33	33/62	0.16
Mean (median) age (years)	69.3 ± 1.6 (71)	67.3 ± 1.3 (71)	0.26
MPO-ANCA	60 (98.3)	70 (73.7)	<0.001
PR3-ANCA	3 (4.9)	15 (15.8)	0.038
Serum creatinine (mg/dl)	1.61 ± 0.23	1.83 ± 0.19	0.45
Disease severity			0.059
Localised	1 (1.7)	3 (3.1)	
Early systemic	21 (34.4)	15 (15.8)	
Generalised	31 (50.8)	60 (63.2)	
Severe	8 (13.1)	17 (17.9)	
General performance^a^			
0/1/2/3/4	11/25/11/12/2	11/33/17/28/6	0.47
BVAS^b^			
BVAS	15.4 ± 1.0	18.4 ± 0.8	0.019
General	49 (80.3)	69 (72.6)	0.27
Cutaneous	13 (21.3)	26 (27.4)	0.39
Mucous membranes/eyes	5 (8.2)	15 (15.8)	0.17
Ear, nose, and throat	9 (14.8)	33 (34.7)	0.006
Chest	22 (36.1)	41 (43.2)	0.38
Cardiovascular	1 (1.7)	11 (11.6)	0.012
Abdominal	0 (0)	2 (2.1)	0.25
Renal	44 (72.1)	65 (68.4)	0.62
Nervous system	28 (45.9)	47 (49.5)	0.66

Values expressed as mean ± standard error or number (percentage) unless otherwise noted. ANCA, antineutrophil cytoplasmic antibody; BVAS, Birmingham Vasculitis Activity Score; ILD, interstitial lung disease; MPO, myeloperoxidase; MPA, microscopic polyangiitis; PR3, proteinase-3. ^a^General performance was categorised according to the World Health Organization Performance Status except category 5 (death). ^b^Disease activity and patterns of organ involvement were defined by the BVAS 2003 scoring system.

## Discussion

This is the first study to apply the EMEA algorithm to prospectively collected and high-quality data of AAV patients outside Europe and to elucidate the clinical phenotypes of the disease. In this study, 156 Japanese patients with newly diagnosed AAV were enrolled from major universities and referring hospitals across Japan and classified according to the EMEA algorithm. The results clearly indicated that MPO-ANCA-positive MPA/RLV was the most common form of AAV in the Japanese population, and more than one-half of the patients with EMEA algorithm-classified GPA showed MPO-ANCA positivity. In addition, we showed that ILD was a common manifestation in Japanese patients with AAV, especially in those with MPA.

The predominance of MPA/RLV and MPO-ANCA positivity in the Japanese population is in marked contrast to the results of studies previously reported from European countries and the United States [3,17-19]. Watts and colleagues validated the EMEA algorithm using 80 paper cases that were originally written for evaluation of the BVAS system for systemic vasculitis with some modifications, representing the relative frequency of AAV in their communities as follows: GPA > MPA > EGPA [4]. It is therefore indispensable and important to evaluate the utility of the EMEA algorithm in ethnicities outside Europe, as we did in this study. We found some difficulties in the classification between GPA and MPA with the EMEA algorithm; for example, of the nine patients classified as having GPA owing to the presence of GPA surrogate markers, five had chronic sinusitis in which granulomatous inflammation was not proven by histology. Because chronic sinusitis is a common disease and because fixed pulmonary infiltrates and otitis media are sometimes observed in AAV diseases other than GPA, classification of AAV using GPA surrogate markers should be cautiously applied in the countries or regions where MPA is more prevalent than GPA.

Within the spectrum of AAV, there are interesting geographic differences in the relative incidence of GPA versus MPA as well as of MPO-ANCA versus PR3-ANCA positivity [20]. In European countries, the incidence of GPA is approximately 4.9 to 10 per million, depending on the geographic location, with higher incidences reported in more northern countries and lower incidences in more southern countries [21,22]. A similar inverse relationship between GPA and MPA has been observed in the Southern Hemisphere [22]. A higher incidence of MPA/RLV than GPA and the predominance of MPO-ANCA found in the Japanese and Chinese AAV populations [3,5] could be related to the lower latitude of these countries.

GPA and MPA are heterogeneous entities with overlapping phenotypes. Recent studies have indicated that the classification system based on ANCA specificity (that is, MPO-ANCA versus PR3-ANCA) may better reflect the phenotypic spectrum of AAV. Cluster analysis of patients with newly diagnosed GPA and MPA from five clinical trials showed that the ANCA specificity classification may be more strongly associated with outcomes such as death and relapse rate than the traditional GPA-MPA separation [23]. Moreover, compared with the CHCC definition and the EMEA algorithm, ANCA specificity was more predictive of relapse in patients with biopsy-proven AAV; patients with PR3-ANCA were almost twice as likely to experience a relapse as those with MPO-ANCA [24]. In this regard, it is intriguing that a genome-wide association study of a European population revealed the presence of genetic distinctions between GPA and MPA that are associated with ANCA specificity [25]. Because of the limited number of patients with PR3-ANCA in our RemIT-JAV cohort, we were not able to perform cluster analysis within this database. We are currently implementing another large-scale cohort study of Japanese patients with AAV, and the combined database will enable us to clarify an association between ANCA positivity and clinical characteristics of AAV in the Japanese population.

MPO-ANCA may contribute to the severity of chronic renal injury and the prevalence of ILD in patients with AAV. Studies of renal biopsy specimens from patients with AAV have demonstrated a higher prevalence and/or severity of renal lesions in MPO-ANCA-positive patients compared with PR3-ANCA-positive patients [26]. These reports are in line with our findings that the mean serum creatinine level of MPO-ANCA-positive patients was numerically higher than that of PR3-ANCA-positive patients (1.94 versus 1.22 mg/dl).

A number of case reports and small case series have indicated that ILD developed more frequently in patients with MPO-ANCA-positive AAV, mainly in those with a diagnosis of MPA, compared with patients with PR3-ANCA-positive AAV [27,28]. A high ratio of MPO-ANCA positivity to PR3-ANCA positivity and a high prevalence of ILD have been reported in Asian countries [8-10], and *vice versa* in northern European countries; ILD was reported in 7.2% of all patients with MPA in the United Kingdom and in less than 10% in other European countries [11,12]. In this study, we confirmed a high prevalence of ILD in Japanese patients with AAV. These patients were categorised as having a milder form (that is, more early systemic and less generalised or severe diseases) and lower disease activity according to the BVAS (Table 5), partially because ILD is not included in these definitions. Investigation of the clinical courses and prognoses of patients with ILD will shed more light on the relevance of ILD in the severity and activity of AAV.

We identified 16 unclassifiable AAV patients with ILD who were eligible for the EMEA algorithm because they were MPO-ANCA-positive, had symptoms and signs compatible with AAV such as general symptoms, and could not be diagnosed as having other diseases. A previous study reported that MPO-ANCA seroconversion from negative to positive occurred in 10% of patients with ILD in their clinical courses and that some patients with MPO-ANCA and ILD eventually developed typical MPA [29]. On the other hand, vasculitis was proven in five of 15 biopsy specimens of MPO-ANCA-positive patients with pulmonary fibrosis [13]. These data indicate that patients with unclassifiable AAV and ILD could be classified as having MPA. Further investigation is required to pursue this possibility.

This study has some limitations. The number of patients evaluated was limited, and the patient data were collected from the university and referral hospitals in large cities in Japan, which might cause tertiary care biases for the relative frequency of AAV diseases.

## Conclusions

MPO-ANCA-positive MPA/RLV is the most common component of AAV in the Japanese population, and more than one-half of patients with GPA are also positive for MPO-ANCA. ILD is an important clinical manifestation in Japanese patients with AAV. Unclassifiable vasculitis with MPO-ANCA positivity and ILD may represent a novel variant of MPA. These data confirm the substantial difference in clinical and ANCA serological features of AAV between western countries and Asian countries, including Japan, and indicate that further investigation and discussion are required from a global perspective for a better AAV classification system that can be applied to all geographic areas and ethnicities.

### Consent

This study was approved by the following ethical committees: Ethics Committee of the Okayama University Graduate School of Medicine, Dentistry and Pharmaceutical Sciences; Medical Research Ethics Committee of Tokyo Medical and Dental University; Kyoto University Ethics Committee Review Board; Ethics Committee of Kagawa University; Ethics Committee of Juntendo University School of Medicine; Ethics Committee University of Tsukuba Hospital; Ethics Committee of St. Marianna University School of Medicine; Kanazawa University Ethical Committee; Ethics Committee of the University of Tokyo; Ethics Committee of Kyorin University School of Medicine; Saitama Medical Center Hospital Ethics Committee; Research Ethics Committee of the University of Miyazaki; Local Ethics Committee of Toho University; Ethics Committee of Kobe University Hospital; Ethics Committee of Kitano Hospital, The Tazuke Kofukai Medical Research Institute; Shimane University Institutional Committee on Ethics; Ethics Review Committee of Nagoya City University Graduate School of Medical Sciences; Ethics Committee of Ehime University Graduate School of Medicine; Ethics Committee of Jichi Medical University; Ethics Committee of Kyoto Prefectural University School of Medicine; Ethics Committee of Tokyo Medical University Hachioji Medical Center; Ethics Committee of Kitasato University Hospital; and Ethics Committee of Hamamatsu University School of Medicine.

## Appendix

Research Committee of Intractable Vasculitis Syndrome of the Ministry of Health, Labour, and Welfare of Japan: in addition to the authors, the following investigators and institutions participated in this study: Department of Rheumatology and Clinical Immunology, Saitama Medical Center, Saitama Medical University (Koichi Amano); Department of Nephrology, Faculty of Medicine, University of Tsukuba (Kunihiro Yamagata); Department of Hemovascular and Artificial Organs, Faculty of Medicine, University of Miyazaki (Shouichi Fujimoto); Department of Respiratory Medicine, Toho University Omori Medical Center (Sakae Homma); Department of Clinical Pathology and Immunology, Kobe University Graduate School of Medicine (Shunichi Kumagai); Center for Nephrology and Urology, Division of Nephrology and Dialysis, Kitano Hospital, Tazuke Kofukai Medical Research Institute (Eri Muso); Department of Rheumatology, Shimane University Faculty of Medicine (Yohko Murakawa); Division of Rheumatology, Department of Medical Oncology and Immunology, Nagoya City University Graduate School of Medical Science (Shogo Banno); Department of Bioregulatory Medicine, Ehime University Graduate School of Medicine (Hitoshi Hasegawa); Division of Nephrology, Department of Internal Medicine, Jichi Medical University (Wako Yumura); Department of Cardiovascular Medicine, Kyoto Prefectural University School of Medicine (Hiroaki Matsubara); Division of Nephrology, Tokyo Medical University Hachioji Medical Center (Masaharu Yoshida); Department of Dermatology, Kitasato University School of Medicine (Kensei Katsuoka); and Third Department of Internal Medicine, Division of Immunology and Rheumatology, Hamamatsu University School of Medicine, Hamamatsu (Noriyoshi Ogawa).

## Abbreviations

AAV: antineutrophil cytoplasmic antibody-associated vasculitis; ACR: American College of Rheumatology; ANCA: antineutrophil cytoplasmic antibody; BVAS: Birmingham Vasculitis Activity Score; CHCC: Chapel Hill Consensus Conference; EGPA: eosinophilic granulomatosis with polyangiitis (Churg–Strauss syndrome); EMEA: European Medicines Agency; GPA: granulomatosis with polyangiitis (Wegener’s granulomatosis); ILD: interstitial lung disease; MPA: microscopic polyangiitis; MPO: myeloperoxidase; PR3: proteinase-3; RemIT-JAV: Remission Induction Therapy in Japanese Patients with ANCA-associated Vasculitides; RLV: renal-limited vasculitis.

## Competing interests

MH has received research grants and/or honoraria from Abbott Japan Co., Ltd, Astellas Pharma Inc., Bristol-Myers Squibb K.K., Chugai Pharmaceutical Co., Ltd, Eisai Co., Ltd, Janssen Pharmaceutical K.K., Mitsubishi Tanabe Pharma Co., Santen Pharmaceutical Co., Ltd, Takeda Pharmaceutical Co., Ltd, Teijin Pharma, Ltd, and Pfizer Japan Inc. TF has received research grants from Abbott Japan Co., Ltd, Astellas Pharma Inc., Bristol-Myers Squibb K.K., Chugai Pharmaceutical Co., Ltd, Daiichi-Sankyo Pharmaceutical Co. Ltd, Eisai Co., Ltd, Mitsubishi Tanabe Pharma Co, Takeda Pharmaceutical Co., Ltd, and Pfizer Japan Inc. HM serves as a consultant to AbbVie Inc., Astellas Pharma Inc., and Teijin Pharma Ltd; received honoraria from Astellas Pharma Inc., MSD K.K., Takeda Pharmaceutical Co., Ltd, and Mitsubishi Tanabe Pharma Co.; and received research funding from Astellas Pharma Inc., Daiichi Sankyo Inc., Dainippon Sumitomo Pharma Co., Ltd, MSD K.K., Novo Nordisk Pharma Ltd, and Takeda Pharmaceutical Co., Ltd.

## Authors’ contributions

KS was responsible for conception and design, data collection and analysis, and manuscript writing. MY, MH, and TF were responsible for conception and design, data collection and analysis, and critical revision. HD, YT, SI, HY, TW, and JH were responsible for data collection and interpretation, and critical revision. YA and HM were responsible for conception and design, data collection and analysis, and critical revision. All authors read and approved the final manuscript.
